# Enhanced extraction of butyric acid under high-pressure CO_2_ conditions to integrate chemical catalysis for value-added chemicals and biofuels

**DOI:** 10.1186/s13068-018-1120-1

**Published:** 2018-04-23

**Authors:** Jaesung Chun, Okkyoung Choi, Byoung-In Sang

**Affiliations:** Department of Chemical Engineering, Hanyang University, 222 Wangshimni-ro, Seongdong-gu, Seoul, 04763 South Korea

**Keywords:** *Clostridium tyrobutyricum*, Butyric acid, Extraction process, Carbon dioxide, High pressure

## Abstract

**Background:**

Extractive fermentation with the removal of carboxylic acid requires low pH conditions because acids are better partitioned into the solvent phase at low pH values. However, this requirement conflicts with the optimal near-neutral pH conditions for microbial growth.

**Results:**

CO_2_ pressurization was used, instead of the addition of chemicals, to decrease pH for the extraction of butyric acid, a fermentation product of *Clostridium tyrobutyricum*, and butyl butyrate was selected as an extractant. CO_2_ pressurization (50 bar) improved the extraction efficiency of butyric acid from a solution at pH 6, yielding a distribution coefficient (*D*) 0.42. In situ removal of butyric acid during fermentation increased the production of butyric acid by up to 4.10 g/L h, an almost twofold increase over control without the use of an extraction process.

**Conclusion:**

In situ extraction of butyric acid using temporal CO_2_ pressurization may be applied to an integrated downstream catalytic process for upgrading butyric acid to value-added chemicals in an organic solvent.

**Electronic supplementary material:**

The online version of this article (10.1186/s13068-018-1120-1) contains supplementary material, which is available to authorized users.

## Background

Short chain fatty acids (SCFA) including butyric acid have potential to be promising platform chemicals for the production of many chemicals and biofuels. Through the chemical catalytic reaction, butyric acid can be converted into hydrocarbons that can be used for the vehicle fuels, such as gasoline, diesel, and jet fuel and for the various application in the fragrance, cosmetic, paint, solvent, and coating industries. Since butyric acid is produced in petrochemical process by chemical synthesis from xxxcrude oils currently, there is a need to produce butyric acid from renewable carbon sources to replace its chemical synthesis and to provide the flexibility needed to accommodate regionally specific biomass [1–3]. In particular, butyric acid can be produced with acetic acid during the acidogenic phase, followed by the solventogenic phase, in Clostridia fermentations [4, 5], and can be converted to the useful platform chemicals, which can be integrated with the existed petrochemical process by chemical catalytic or enzymatic esterification, putative enzymatic decarboxylation, and catalytic decarboxylation [6].

Butyric acid production with fermentation is one of the oldest and most-studied processes, and various genera have been investigated for their feasibilities of industrial application. *Clostridium tyrobutyricum*, *Clostridium acetobutyricum*, *Clostridium thermobutyricum* are some of the most generally investigated and industrially used strains [7]. *C. tyrobutyricum* has been the preferred strain for butyric acid production [8, 9], and produced 55.2 g/L of butyric acid with 3.22 g/L/h of productivity using pretreated molasses [10] and 58.8 g/L with a productivity of 1.9 g/L/h using a combination of sweet sorghum stalks and beet molasses [11]. For the industrial scale production of butyric acid, separation and recovery technology of butyric acid from fermentation broth is recognized as a major challenge due to the process operation technological hurdles, but also due to product inhibition of butyric acid from fermentation broth by the toxicity of butyric acid at relatively low concentrations [12]. To be concentrated from the fermentation broth, butyric acid can be recovered by the use of nanofiltration membrane, liquid–liquid extraction, electrodialysis [13], and adsorption [14]. Since the contribution of downstream processing costs including the separation and recovery technologies is typically 30–40% of the total production costs, development of a competitive separation and recovery process is important to enable microbial production of butyric acid [15]. Type of inorganic acid or base to adjust the optimum pH for butyric acid production is also considered and these may determine how process steps can be integrated, how side streams may be reused in the process, and which separation and recovery processes can actually be used. Therefore, separation and recovery processes are required in a biorefinery to separate and purify the products and intermediates for the next stage of processing such as chemo-catalytic conversion for value-added chemical or fuel production, and to remove the inhibitory effects of butyric acids produced during fermentation. For in situ product recovery of butyric acid from fermentation broths, extractive fermentation has been attempted, and adsorption and extraction showed fairly good performance in the continuous acid recovery from anaerobic fermentation [16–18].

In the extractive fermentation, protonated species of butyric acid at low pH values below its pKa of 4.82 at 25 °C improve its extraction efficiency. However, this requires the use of cyclic pH changes to transition between optimal microbial growth conditions (pH 6–7) and partitioning into the solvent phase (pH < 4). The addition of acid to the downstream partitioning process causes the accumulation of ions in the culture medium and hinders microbial growth by increasing the osmotic stress on bacteria [16, 19].

CO_2_ sparging has been used to achieve temporarily lower pH values for enhancing performance of the extractive fermentation without leaving the accumulation of salts from the large additions of acid and base for pH shifts and without expense of fermentation productivity. Elevated CO_2_ pressures with repeated 1-h cyclical exposure up to 60 bar of pCO_2_ result in more effective pH swings (up to pH 3.8 in 5 g/L yeast extract) compared with atmospheric CO_2_ sparging without having an inhibitory effect on *C. tyrobutyricum* [16, 20].

**Fig. 1:**
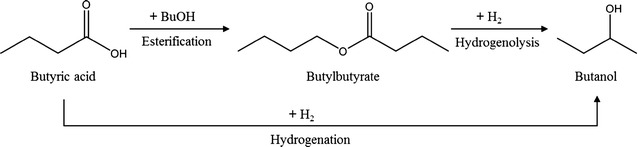
Pathway used to upgrade butanol from butyric acid

Recent efforts have been made to upgrade fermentation products to value-added chemicals and to further integrate chemical catalysis with extractive fermentation [6, 21–23]. One such attempt is the use of a transition-metal catalyst for alkylation in ABE fermentation for conversion to a higher-molecular-mass fuel [21]. Another is the upgrading of butyric acid to butanol by hydrogenation [24] or by esterification [25, 26], and hydrogenolysis [27–29] (Fig. 1).

To operate a continuous process integrated with extractive fermentation and catalytic process, the properties of extractant in the extractive fermentation should be considered for downstream processes such as catalytic upgrading of a fermentation product to value-added chemicals. For example, tertiary amines are easily extractible but, due to its corrosive nature and high reactivity with chemical catalysts, it requires special attention as extractants may not react with catalysts in future steps. We also considered the process by which butyric acid is integrated with value-added chemicals by investigating the use of CO_2_-mediated pH swings, and chose an extractant for liquid–liquid extraction. Butyl butyrate was selected as an extractant for butyric acid because it is not an amine-type chemical or a corrosive substance that could react with the catalyst downstream [30].

Here, we show for the first time the use of high CO_2_ pressure for the liquid–liquid extraction of butyric acid from fermentation medium. While a previous study of the application of high CO_2_ pressure was conducted through direct absorption between cells and polymeric absorbents [20], this study used the solvent extraction under high CO_2_ pressure and minimized microorganism toxicity of solvent by separation of the cells and the extraction process through cell recovery through the membrane. The aim of this study was to investigate the increase of butyric acid extraction efficiency in liquid–liquid extraction through a temporary decrease in pH using high CO_2_ partial pressure.

## Results and discussion

### Extraction of butyrate using butyl butyrate under high pCO_2_

Butyl butyrate was selected from among oleyl alcohol, dodecanol, and mixtures of trioctylamine or ditridecylamine as a solvent for the extraction of butyric acid from the fermentation medium because it is not an amine-type chemical, nor a corrosive substance that can react with the catalyst downstream during the conversion of butyrate into various chemicals and fuels [30] (refer to Additional file 1: Figure S1 for the different extraction efficiencies of solvents). If amine-type solvents are used as extractants, it is necessary to remove the extractant before the catalytic process, which incurs an additional cost and integrates as a continuous process extractive fermentation and the catalytic process.

**Fig. 2:**
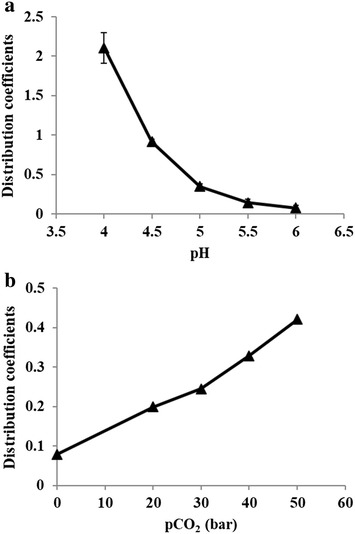
The effect of pH on extraction using butyl butyrate as an extractant. **a** Distribution coefficients of butyric acid vs. initial pH in the aqueous phase, **b** distribution coefficients of butyric acid vs. CO_2_ partial pressure

The distribution coefficient (*D*) of butyrate using butyl butyrate was found to be dependent on pH; the distribution coefficient increased up to 2.11 ± 0.19% at pH 4.0, from 0.08 ± 0.03% at pH 6.0 (Fig. 2a), due to an increase in undissociated acid forms as pH values decrease below the pKa of butyric acid (4.8). The dependence of distribution coefficients on CO_2_ partial pressure is shown in Fig. 2b. From the distribution coefficients in both Fig. 2a, b, the pH value under 50 bar CO_2_ in liquid extraction with an equal volume of butyl butyrate can be inferred as approximately as 4.9 because the distribution coefficients at 50 bar is 0.42, similar to the expected value at pH 4.9.

From Henry’s law (Eq. 1), the concentration of CO_2_ dissolved in water at 50 atm of pressure and 298.15 K is 1.7 M.

1 $$C_{\text{aq}} = kP_{\text{g}}$$

The Henry’s law constant, *k* is 3.4 × 10^−2^ mol L^−1^ atm^−1^ of CO_2_ in water at 298.15 K, and *P*_g_ is the partial pressure of gas. The first acid equilibrium of CO_2_ is predominant and to account for the fact that CO_2_ (aq) is in equilibrium with H_2_CO_3_ (aq) and that the proton and bicarbonate concentrations are equal,

2 $$K_{{{\text{A}}1}} = \frac{{\left[ {{\text{H}}^{ + } } \right]\left[ {{\text{HCO}}_{3}^{ - } } \right]}}{{\left[ {{\text{CO}}_{2} \left( {\text{aq}} \right)} \right]}} = \frac{{\left[ {{\text{H}}^{ + } } \right]^{2} }}{{\left[ {{\text{CO}}_{2} \left( {\text{aq}} \right)} \right]}} = 4.45 \times 10^{ - 7}$$

From Eqs. 1 and 2, the pH is 3.06 at 50 atm, the partial pressure of CO_2_ (pCO_2_). The reason the pH value under these conditions is higher than 3.06, the calculated pH at pCO_2_ 50 atm (50.7 bar), is the buffering effect of medium (Additional file 1: Figure S3). The buffering effects of medium components such as yeast extract and phosphate have been observed in previous studies [20, 31]. Ammonium acetate present in the medium seems to strongly resist pH changes, and the filtrate of culture broth exhibited the strongest buffering capacity; a change in pH of ∆pH = 0.08 was found under CO_2_ purging at ambient pressure (Additional file 1: Figure S3). When the amount of the butyl butyrate is twice the volume of the culture medium, the extraction efficiency of butyrate using butyl butyrate under 50 bar CO_2_ was 45.7% (data not shown). Previous studies have shown that the removal of butyric acid by polyether Pebax 2533 (solid–liquid extraction) improves from 3 to 40% upon acidifying a pH 6 solution with 60 bar of CO_2_ [32]. The high measured extraction efficiency of butyrate in our study indicates that the use of high pCO_2_ is more efficient in the liquid–liquid phase rather than in the solid–liquid phase extractions.

### The extractive fermentation of *C. tyrobutyricum*

**Fig. 3:**
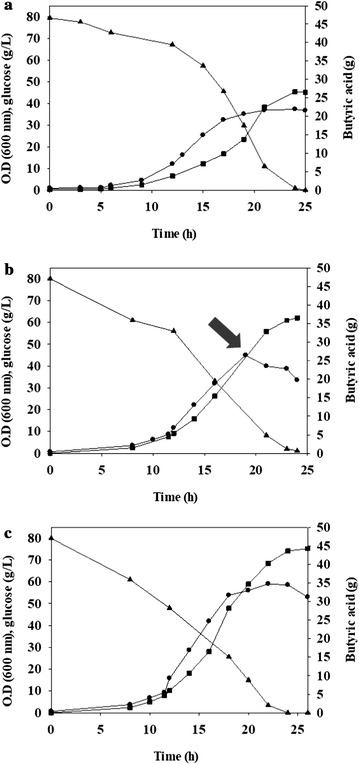
Microbial growth (circle, represented as the optical density at 600 nm), glucose consumption (triangle), and butyric acid production (square) without extraction (**a**), and with CO_2_-pressurized (50 bar) extraction (**b**, **c**). The arrow indicates the toxicity of butyl butyrate remaining in the aqueous phase (**b**). After the removal of residual butyl butyrate using tetradecane, both glucose consumption rate and butyric acid productivity increased (**c**)

**Fig. 4:**
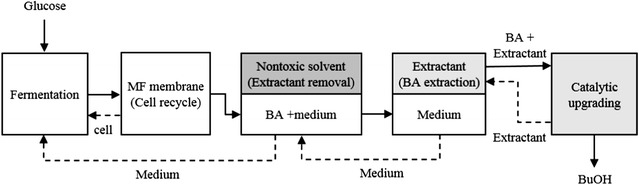
Experimental setup for fermentation and CO_2_-pressurized extraction processes. Dashed lines represent the proposed recovery operation for the removal of butyl butyrate (BB) with tetradecane. Butyric acid (BA) produced by *C. tyrobutyricum* was extracted using a CO_2_-pressurized liquid–liquid extraction system after microfiltration (MF)

A high pCO_2_ was used for the extraction of butyric acid, a glucose fermentation product produced from *C. tyrobutyricum.* The filtrate produced from microfiltration was used for butyric acid extraction. Figure 3 shows the time profile of microbial growth and the concentration changes of glucose (substrate) and butyric acid (product) without (Fig. 3a) or with (Fig. 3b, c) liquid–liquid extraction using high pCO_2_ (CO_2_ pressure–liquid extraction). The total amount of butyric acid produced through CO_2_ pressure–liquid extraction is the sum of butyric acid in the aqueous and organic phases. The rate of glucose consumption and butyric acid production increased compared with that produced without extraction (Fig. 3a, b). However, the optical density decreased after approximately 20 h of incubation under CO_2_-pressurized liquid extraction (Fig. 3b). This phenomenon is caused by residual butyl butyrate in the aqueous phase of approximately 1.9 g/L, as measured using GC-FID (for more detail see Additional file 1: Figure S4). As a result, butyl butyrate induced microbial death, resulting in a decrease in optical density. Due to the decrease of microbial growth, production of butyric acid also decreased. The toxicity of butyl butyrate on microbial growth of 1 g/L was measured (Additional file 1: Figure S4). Therefore, an extraction reservoir was used to remove residual butyl butyrate from the aqueous phase (Fig. 4). Tetradecane was used to prevent the inflow of butyl butyrate to the aqueous phase. Figure 2c shows the increase of microbial growth associated with a higher rate of glucose consumption (5.55 g/L h) and butyrate production (3.99 g/L h) after tetradecane treatment.

**Fig. 5:**
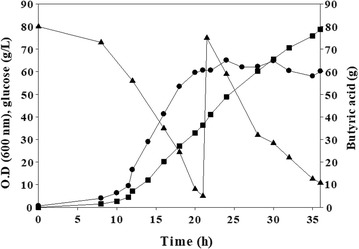
Fed-batch fermentation with CO_2_-pressurized (50 bar) liquid–liquid extraction. Microbial growth (circle, represented as the optical density at 600 nm), glucose consumption (triangle), and butyric acid production (square) are shown, and butyric acid productivity was 4.10 g/L h

The kinetic parameters for each condition are summarized and compared in Table 1. The results of extractive fermentation under 50 bar pCO_2_ were divided into two conditions; without/with the removal of butyl butyrate from the aqueous phase. The final titer of butyrate was changed after CO_2_ pressure–liquid extraction and was measured at 27.4 g under control conditions vs. 36.5/45.1 g under extractive fermentation conditions at 50 bar pCO_2_ (Table 1). However, the productivity of butyric acid increased from 2.3 up to 3.99 g/L h. Previous studies of solid–liquid phase extraction under 60 bar showed that the productivity was decreased (0.44 vs. 0.50 g/L h), while the final titer was increased (74 vs. 68.4 g) in the batch system [20]. The productivities reported in previous studies of *C. tyrobutyricum* are shown in Table 2. The productivity in fed-batch fermentation was below 2 g/L h. The value we measured was slightly higher, 2.30 g/L h, and after extractive fermentation under 50 bar pCO_2_, it increased to 3.99 g/L h (Table 1). Fed-batch fermentation was performed with two glucose feedings (each 80 g/L) (Fig. 5). Total butyrate production was 80.9 g, and the productivity of butyrate was 4.10 g/L h, which are comparable to the values measured in previous studies (Table 2). The increased production of butyrate has been demonstrated in studies of endproduct inhibition in *C. tyrobutyricum* [33]. The yield of butyrate is relatively constant (~ 0.3 g/g), but the production of butyrate increased at higher dilution rates [33, 36]. The in situ extraction of butyric acid increased the production rate (productivity) of butyric acid generated by *C. tyrobutyricum*.

**Table 1:** Comparison of butyrate production and extraction efficiency in batch fermentation

	Batch fermentation		
	Control	Liquid–liquid extraction under 50 bar pCO_2_	
		Without the recovery of butyl butyrate from the aqueous phase	With the recovery of butyl butyrate from the aqueous phase
Butyric acid production (g)	27.4	36.5	45.1
Extraction efficiency (%)	–	62.1	57.8
Butyric acid productivity (g/L h)	2.30	3.39	3.99
Y (g butyric acid/g glucose)	0.38	0.30	0.38

**Table 2:** Comparison of butyrate productivity in extractive fed-batch fermentation with previous studies of *C. tyrobutyricum*

	Productivity (g/L h)	References
Fed-batch fermentation	0.82	Michel-Savin et al. [33]
	1.25	Fayolle et al. [34]
	1.41	Song et al. [35]
	1.9	Sjöblom et al. [11]
Extractive fermentation	2.15	Du et al. [18]
	7.37^a^	Wu and Yang [17]
	4.10^b^	This study

^a^Immobilized cells of *C. tyrobutyricum*

^b^Extractive fed-batch fermentation

The upgrading of carboxylic acids to their corresponding aldehydes, alcohols and hydrocarbons require carefully balanced oxygen removal reactions, such as several catalytic routes including dehydration, hydrogenolysis and hydrogenation [37]. For the production of larger molecules appropriate for diesel and jet fuels, C–C coupling reactions such as ketonization or esterification reactions can also be exploited [25, 38, 39]. In addition, butyl butyrate produced by esterification with butyric acid can be used for the production of butanol by hydrogenolysis with hydrogen [27]. The intermediate step of esterification allows milder conditions to be used compared to direct catalytic conversion to butanol. Subsequent hydrogenolysis of butyl butyrate to butanol was tested with a commercially available Cu/ZnO/Al_2_O_3_ catalyst prepared with 1.0 wt.% palladium under a downstream reactor pressure of 10 bar, temperature of 150–200 °C [28]. After hydrogenolysis, one part of the butanol is then used as a product and one part is used for the esterification reaction to produce butyl butyrate. By associating the above process with our work, the biohydrogen produced during the fermentation as well as the use of butyl butyrate for the extraction of butyric acid could in principle be used for the hydrogenolysis reaction making the process more sustainable. Therefore, when the extractive fermentation with butyl butyrate for butyric acid production is used under 50 bar pCO_2_, the catalytic upgrading of butyric acid with particular focus on butanol as a target product is not required to supply the additional pressure and solvents. In order for catalytic upgrading to be commercially applicable, efficient recovery processes of the carboxylic acid in combination with cost-effective catalytic systems must be developed. Integrated recovery and upgrading systems with butyl butyrate under high CO_2_ pressure are highly attractive and minimize waste and energy consumption.

## Conclusions

CO_2_ pressure–liquid extraction increased the extraction efficiency of butyric acid from a culture broth of *C. tyrobutyricum*. The extraction efficiency was higher (62.1%) than that previously found in studies of CO_2_ pressure–solid phase extraction. The removal of butyric acid through extractive fermentation led to an increase in the productivity of butyric acid from 2.30 to 3.99 g/L h, and reached 4.10 g/L h through fed-batch fermentation. CO_2_ pressure–liquid extraction demonstrated a high extraction efficiency for butyric acid and made possible an integrated catalytic process with extractive fermentation to upgrade butyric acid to a value-added chemical downstream with the selection of an appropriate solvent.

## Methods

### Bacterial strains, medium, and materials

*Clostridium tyrobutyricum* ATCC 25755 was cryopreserved with 25% glycerol at − 78 **°**C until use and cultivated in serum bottles sealed with rubber stoppers and aluminum crimp seals. We modified P2 medium [40] for use as a fermentation medium with 80 g/L of glucose and 25 g/L of yeast extract (BD Difco, Sparks, MD). To cultivate anaerobic conditions, the medium was purged with argon gas (99.9%) for 30 min and autoclaved prior to use. The pH of the medium was initially adjusted to 7.0 using 3.0 N NaOH and controlled at 6.0, the optimal pH for the production of butyric acid by *C. tyrobutyricum* during fermentation [10]. The Cultivation temperature was 37 °C. Butyl butyrate and tetradecane were purchased from Kasei Kogyo Co., Ltd. (TCI, Tokyo, Japan). All chemicals were of analytical or HPLC grade and used without further purification.

### Extraction of butyrate using butyl butyrate under high pCO_2_

The dependence of distribution coefficient on pH was preliminarily tested under various pH values of 4, 4.5, 5, 5.5, 6, and 6.5. The pH was adjusted using 3 M HCl. The distribution coefficient (*D*) was calculated as per Eq. 3.

3 $$D = \frac{{C_{\text{sol}} }}{{C_{\text{aq}} }}$$

Here, *C*_sol_ is the concentration of butyric acid presented in the solvent phase, and *C*_aq_ is the concentration in the aqueous phase (fermentation broth) after extraction.

To verify the effect of increased CO_2_ pressure on butyrate extraction from medium, 150 and 300 mL of butyl butyrate were added to 150 mL of filtrated fermentation broth in a 1-L stainless vessel equipped with agitation, temperature, and pressure gages. The vessel was continuously pressurized at 20, 30, 40, and 50 bar of CO_2_ and agitated at 500 rpm for 10 min. The final aqueous concentration of butyric acid was analyzed to calculate the extraction efficiency. The extraction time did not affect the extraction efficiency and did not exhibit significant changes after 10 min of mixing (Additional file 1: Figure S2).

### The extractive fermentation of *C. tyrobutyricum*

Figure 4 illustrates the processes and equipment used in this study. Fermentation was performed by connecting a flat type membrane and a hydrophilic PVDF microfiltration (MF) module (0.45 μm, 0.1 m^2^, Millipore, USA) for cell recovery. All cultures were grown anaerobically at 37 °C, 150 rpm. The pH was initially set at 7 and controlled at pH 6 after inoculation. Batch or fed-batch fermentation was initially conducted in 3-L fermenter with a 1.5-L working volume. For preculture, 100 mL stock cultures were used to inoculate 75 mL of P2 medium for about 12 h.

Bioreactors were arranged sequentially and performed fermentation, cell recycling, and butyric acid extraction functions. For the removal of butyl butyrate, one reservoir was prepared with tetradecane (Fig. 4). The assembly used consisted of three or four jacketed bioreactors treating a fermentation culture volume of 1.5 L. The bioreactor was inoculated with 5% *C. tyrobutyricum*. Fermentation was allowed to proceed in batch mode for 12 h, and both cell recycling and extraction were begun. Culture broth was circulated at 20 mL/min, keeping 900 mL working volume in the fermenter, 300 mL in the reservoir, and 300 mL in the extractor. Cells were recovered by the membrane module as a rate of 400 mL filtrate/min using microfiltration feeding at 500 mL culture-broth/min.

A 1-L extraction vessel containing 600 mL of butyl butyrate (extractant) was used to remove butyrate generated during continuous cultivation from fermentation. The extraction vessel was pressurized with CO_2_ at 50 ± 5 bar. Culture broth (300 mL) and extractant (600 mL butyl butyrate) were mixed by continuous agitation at 100 rpm, and the extractor volume was maintained at 900 mL. The flow rate of output from the extractor was 20 mL/min. The pressure of the extractor was regulated by CO_2_ bombe and a back-pressure valve.

A reservoir was prepared with tetradecane (200 mL) for the removal of butyl butyrate from the medium to prevent its introduction into the medium. The reservoir was agitated at 100 rpm to allow full mixing. The working volumes of all of the bioreactors used were kept constant by removing extra medium with peristaltic pumps.

To operate fed-batch fermentation, additional glucose and yeast extract were added intermittently to the culture using a concentrated solution; when the glucose level fell below 5 g/L, it was replaced to adjust the initial concentration of glucose (80 g/L) and other medium components, using a 300 mL bolus. At the same time, 600 mL of butyl butyrate in the extractor was replaced for the treatment of a second batch of fermentation.

In the extractive fermentation, extraction efficiency was calculated as shown in Eq. 4.

4 $${\text{Extraction}}\;{\text{efficiency}}\;\left( \% \right) = \frac{{{\text{Butyric}}\;{\text{acid}}\;{\text{in}}\;{\text{the}}\;{\text{solvent}}\;{\text{phase}}}}{{{\text{Total}}\;{\text{butyric}}\;{\text{acid}}\;{\text{in}}\;{\text{the}}\;{\text{extraction}}\;{\text{vesssel}}}}$$

The solvent in the extraction vessel was sampled by bellows valve, and the amount of butyric acid in the aqueous phase was analyzed from fermentation broth leaving the vessel after extraction. The productivity was calculated from the 900 mL working volume in the fermenter at the late exponential phase.

### Analytical method

Cell concentrations were estimated by optical density OD, at 600 nm. Butyric acid in acidified samples with 100 mM phosphoric acid was analyzed using gas chromatography (Agilent Technologies, Model 7890, Palo Alto, CA, USA) equipped with a flame ionization detector (FID), a 30 m × 0.25 μm × 0.25 μm HP-INNOWAX column and nitrogen as carrier gas. The concentration of glucose was reflectometrically measured using an RQflex 10 reflectometer (Merck Inc.).

## Additional file

**Additional file 1: Figure S1.** The different extraction efficiencies of solvents used for butyric acid extraction. **Figure S2.** A comparison of extraction efficiencies at different extraction times. **Figure S3.** pH changes after CO_2_ purging. **Figure S4.** Microbial growth in fresh medium including 1 g/L butyl butyrate with or without tetradecane treatment.
