# Supplementary material for: Enhanced extraction of butyric acid under high-pressure CO2 conditions to integrate chemical catalysis for value-added chemicals and biofuels
Source: Biotechnol Biofuels. 2018 Apr 23;11:119. doi: 10.1186/s13068-018-1120-1 (PMC5911967; doi:10.1186/s13068-018-1120-1)
Supplement: Supplementary file 1 — Additional file 1: Figure S1. The different extraction efficiencies of solvents used for butyric acid extraction. Figure S2. A comparison of extraction efficiencies at different extraction times. Figure S3. pH changes after CO2 purging. Figure S4. Microbial growth in fresh medium including 1 g/L butyl butyrate with or without tetradecane treatment. [file 13068_2018_1120_MOESM1_ESM.docx]

Additional file 1

**Enhanced extraction of butyric acid under high pressure CO_2_ conditions to integrate chemical catalysis for value-added chemicals and biofuels**

Authors:
Jaesung Chun, Okkyoung Choi, and Byoung-In Sang^*^

Affiliation:
Department of Chemical Engineering, Hanyang University, 222 Wangshimni-ro, Seongdong-gu, Seoul 04763, South Korea

Number of pages: 7

Number of figures: 4

**Fig. S1.** The different extraction efficiencies of solvents used for butyric acid extraction.

**Fig. S2.** A comparison of extraction efficiencies at different extraction times.

**Fig. S3.** pH changes after CO_2_ purging.

**Fig. S4.** Microbial growth in fresh medium including 1 g/L butyl butyrate with or without tetradecane treatment.

**
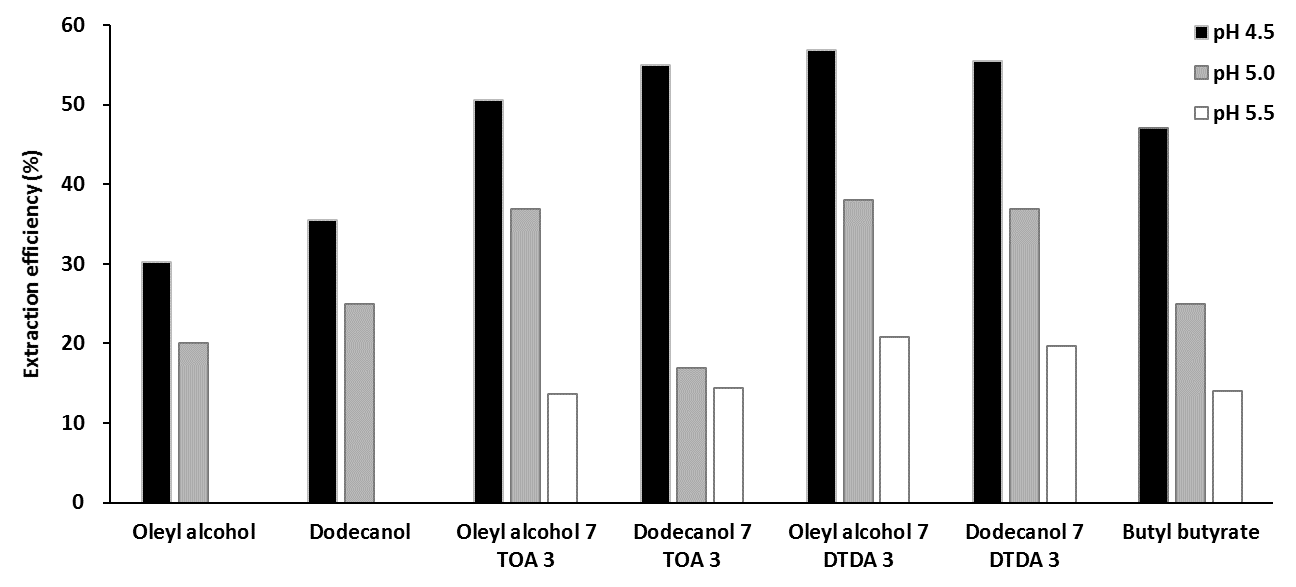
**

**Fig. S1.** The different extraction efficiencies of solvents used for butyric acid extraction. Equal volumes of solvent were used and fully agitated at 700 rpm under different pH conditions: 4.5, 5.0, or 5.5. DTDA (ditridecylamine) and TOA (trioctylamine) were each used as a mixture with oleyl alcohol or dodecanol at the indicated ratio. The extraction efficiency was calculated as following: extraction efficiency, % = butyric acid extracted / total butyric acid x 100.


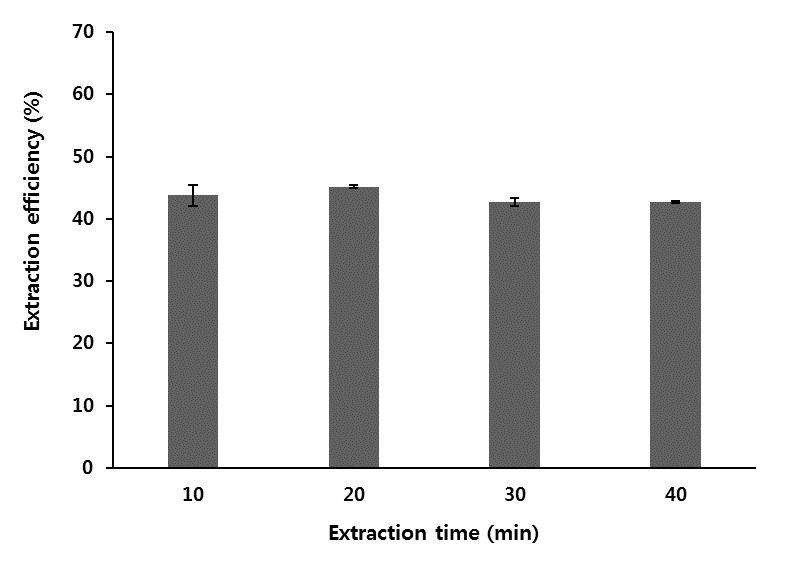


**Fig. S2.** A comparison of extraction efficiencies at different extraction times. The results show no large differences after 10 min of mixing. The extraction efficiency was calculated as following: extraction efficiency, % = butyric acid extracted / total butyric acid x 100.

**The buffering effect of medium**

Resistance to pH changes in different media was tested. Tests were performed under ambient pressure and room temperature. The pH was not adjusted, and reactor was sparged with carbon dioxide at 2-3 L/min and agitated (150 rpm) for gas mixing. The pH change (△pH) was determined by subtracting the final pH from the initial pH. Ammonium acetate exhibited the largest effect on pH resistance (Fig. S1). The pH was more resistant to change in broth after fermentation than in medium (Fig. S1). The production of extracellular components such as proteins during fermentation appears to contribute to resistance to pH changes.

Modified P2 medium (mP2) was prepared based on P2 medium (Qureshi and Blaschek 1999). The amounts of glucose and yeast extract used were modified in mP2.

1. mP2 medium components (g/L)

1.1. Acetate buffer

KH_2_PO_4_ 0.5

K_2_HPO_4_ 0.5

Ammonium acetate 2.2

1.2. Vitamins

Para-amino-benzoic acid 0.001

Thiamine 0.001

Biotin 0.00001

1.3. Minerals

MgSO_4_ 0.2

MnSO_4_ 0.01

FeSO_4_ 0.01

NaCl 0.01

**
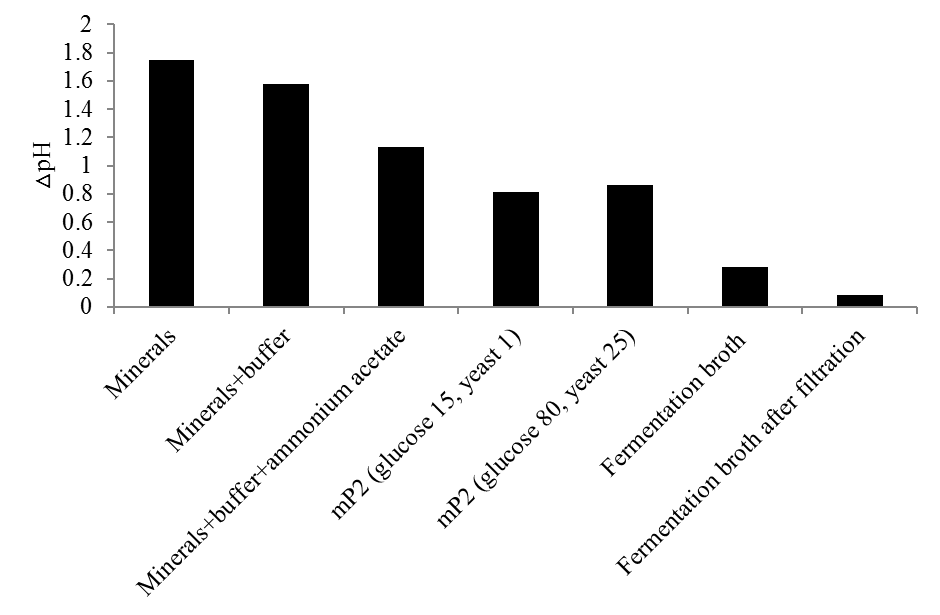
**

**Fig. S3.** pH changes after CO_2_ purging. The pH change is calculated by subtracting the final pH from the initial pH for each condition.

**The toxic effect of butyl butyrate on microbial growth**

A toxicity test was performed by observing microbial growth after the injection of butyl butyrate. After 10 hours of incubation in fresh medium, three different mediums were added. Fresh medium was added as a control. And the medium containing 1 g/L butyl butyrate was added to another. Tetradecane-treated medium was added to the other after mixing medium including 1 g/L butyl butyrate with tetradecane for 5 min. Butyl butyrate inhibited microbial growth (Fig. S4). Tetradecane removed butyl butyrate from the aqueous medium, and microbial growth completely recovered after tetradecane treatment (Fig. S4).

**Fig. S4.** Microbial growth in fresh medium including 1 g/L butyl butyrate with or without tetradecane treatment. Lower levels of growth indicate the microbial toxicity of butyl butyrate. After mixing fresh medium including butyl butyrate with tetradecane, the residual butyl butyrate in the medium was removed and the microbial growth was observed to be similar to that in the control.

**References**

Qureshi N, Blaschek HP. 1999. Butanol recovery from model solution/fermentation broth by pervaporation: evaluation of membrane performance. Biomass and Bioenergy 17(2):175-184.
